# *In-situ* composite microstructure formation of immiscible alloy solidified in space

**DOI:** 10.1093/nsr/nwac261

**Published:** 2022-11-17

**Authors:** Jiuzhou Zhao, Hao Sun, Lili Zhang, Hongxiang Jiang, Linjie Yang, Jie He

**Affiliations:** Shi-changxu Innovation Center for Advanced Materials, Institute of Metal Research, Chinese Academy of Sciences, China; School of Materials Science and Engineering, University of Science and Technology of China, China; Shi-changxu Innovation Center for Advanced Materials, Institute of Metal Research, Chinese Academy of Sciences, China; School of Materials Science and Engineering, University of Science and Technology of China, China; Shi-changxu Innovation Center for Advanced Materials, Institute of Metal Research, Chinese Academy of Sciences, China; School of Materials Science and Engineering, University of Science and Technology of China, China; Shi-changxu Innovation Center for Advanced Materials, Institute of Metal Research, Chinese Academy of Sciences, China; School of Materials Science and Engineering, University of Science and Technology of China, China; Shi-changxu Innovation Center for Advanced Materials, Institute of Metal Research, Chinese Academy of Sciences, China; School of Materials Science and Engineering, University of Science and Technology of China, China; Shi-changxu Innovation Center for Advanced Materials, Institute of Metal Research, Chinese Academy of Sciences, China; School of Materials Science and Engineering, University of Science and Technology of China, China

## Abstract

The immiscible alloy Ti-Co-Gd is solidified in space by using the Electrostatic Levitation Device on board the Chinese Space Station. A sample with *in-situ* composite structure is obtained. The microstructure formation and gravity effect are discussed.

About one-third of alloys show a phase diagram with a miscibility gap in the liquid state. The solidifications of these alloys proceed with a liquid-liquid decomposition and have great potential in the development of high-performance *in-situ* particulate composites, and composites with a core/shell structure. However, these alloys generally show a phase segregated microstructure when solidified on the ground. Since the 1960s, solidification experiments have been done under microgravity conditions in space to circumvent gravity effects. But most of them failed in obtaining an expected composite microstructure [1]. Past research demonstrated that the cooling rate and the surface contamination from the crucible have a great effect on the solidification of immiscible alloys [1–3]. Container-less solidification in space can achieve a rapid or sub-rapid cooling rate and avoid surface contamination, and thus is favorable for investigation of the solidification of immiscible alloys. But no such experiments have been carried out in space to date.

In this research the Ti-35wt%Co-15wt%Gd immiscible alloy was solidified in space by using an Electrostatic Levitation Device (ELD) on board the Chinese Space Station (CSS), as shown in Fig. 1A. The experiments were done under a vacuum environment, ∼10^−3^ Pa. The experimental procedure is: levitate an alloy ball of 2.7 mm in diameter and melt it using four laser beams first, and maintain a holding temperature of ∼1500°C for 2 min; then solidify the alloy byswitching off the power of the laser beams. The alloy ball experienced a cooling rate of ∼111°C/s during cooling in the miscibility gap, as shown in Fig. 1B (μg-S). For comparison, solidification experiments were carried out on the ground using the same ELD, following the same procedures but using a smaller alloy ball of 1.7 mm in diameter due to the limitation of the levitation ability of the ELD on the ground. The alloy ball experienced a higher cooling rate of ∼149°C/s, as shown in Fig. 1B (1g-S).

**Figure 1.**
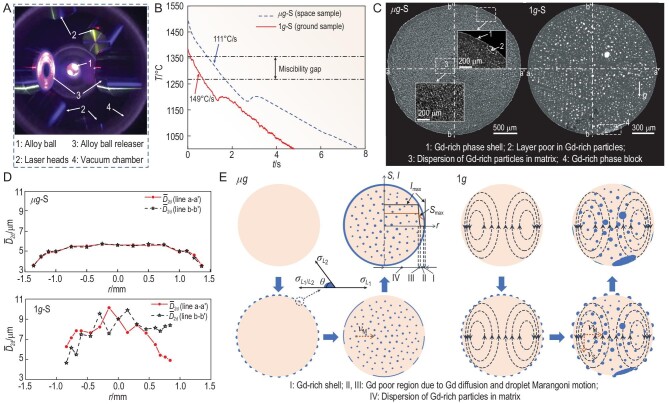
Solidification microstructure formation of Ti-Co-Gd alloy. (A) Electrostatic Levitation Device. (B) Cooling curves of samples. (C) Microstructure of the samples. (D) Average two-dimensional diameter of Gd-rich particles $\bar{D} $_2d_ along the lines shown in Fig. 1C. r is the radial position. (E) Schematics of the microstructure formation during liquid-liquid decomposition in space and on the ground. *I*_max_ and *S*_max_ are the maximum nucleation rate of the Gd-rich droplets and the maximum supersaturation of solute Gd in the matrix liquid as a function of *r*. $\theta $ is the contact angle between the ball surface and the Gd-rich droplets. *v*_M_ and *v*_S_ are the Stokes and Marangoni motion velocity of droplets, respectively.

The microstructure of the space sample μg-S consists of a very thin surface shell of the Gd-rich phase, a Gd-rich-phase poor region next to the shell and a well dispersed microstructure inside, as shown in Fig. 1C and D. In contrast, although cooled at a higher rate, ground sample the 1g-S shows a microstructure with much coarser Gd-rich particles, and a big block of the Gd-rich phase appears on its surface (see Fig. 1C and D), indicating that phase segregation has occurred to some extent. The differences between the microstructures of the two samples are caused by the gravity-related convective flow of the melt and the Stokes motion ${v}_{\rm{S}}$ of the Gd-rich droplets, as shown in Fig. 1E. The densities of the Gd-rich droplets and the (TiCo)-rich matrix liquid vary respectively in the ranges of 7318∼7394 kg/m^3^ and 5980∼5952 kg/m^3^ during the cooling of the alloy through its miscibility gap. When solidifying the alloy on the ground, the Gd-rich droplets sink in the matrix melt and the natural convectionoccurs in the matrix melt. It is the Stokes motion of the Gd-rich droplets together with the natural convection that promotes the collisions and coagulations between the Gd-rich droplets and leads to a rapid coarsening of the droplets, causing the formation of a phase-segregated microstructure, as shown in Fig. 1E. The almost-spherical symmetry of the solidification microstructure of the μg-S (see Fig. 1C and D) indicates that the convection in the melt and the Stokes motion of the Gd-rich droplets are negligibly weak and a temperature field of approximately spherical symmetry has been achieved when solidifying the alloy in space. The microstructure formation in space is relatively simple. It is a result of the concurrent actions of the nucleation, diffusional growth and the ball center-towards Marangoni migration ${v}_{\rm{M}}$ of the Gd-rich droplets. The surface energies of the pure Ti, Co and Gd at their melting temperatures are respectively ${\sigma }_{{\rm{Ti}}} = 1.65$, ${\sigma }_{{\rm{Co}}} = 1.873$ and ${\sigma }_{{\rm{Gd}}} = 0.81\,{\rm{J}}{{\rm{m}}}^{ - 2}$ [4]. Calculations using the model and thermodynamic data [5,6] indicate that the interfacial energy between the two liquid phases ${\sigma }_{{L}_1{L}_2}$ varies between $0.031{-}0.051\,{\rm{J}}{{\rm{m}}}^{ - 2}$ in the miscibility gap. It can be deduced based on these data that ${\sigma }_{{L}_1{L}_2}$, the surface energies of the TiCo-rich matrix liquid $( {{\sigma }_{{L}_1}} )$ and the Gd-rich liquid $( {{\sigma }_{{L}_2}} )$ satisfy ${\sigma }_{{L}_1} > {\sigma }_{{L}_2}$ and ${\sigma }_{{L}_2} + {\sigma }_{{L}_1} > {\sigma }_{{L}_1{L}_2}$. Thus, the contact angle $\theta $ between the ball surface and the Gd-rich droplets is below $\pi /2$ (see Fig. 1E) and the ball surface has a catalyzing effect on the nucleation of the Gd-rich droplets. Compared to the homogeneous nucleation inside the ball, the heterogeneous nucleation of the Gd-rich droplets occurs a little earlier on the ball surface at a lower supersaturation, but at a high rate. It causes the much higher local number density of the Gd-rich droplets. The nucleation and diffusional growth of the droplets lead to a quick drop in local supersaturation, and thus a diffusional transfer of solute Gd towards the ball surface. As a result, the adjacent region of the ball surface (region II in Fig. 1E) can only achieve a lower supersaturation and, therefore, a lower or even 0 nucleation rate and number density of the Gd-rich droplets, as shown schematically in Fig. 1E. It is this diffusional transfer of solute Gd which caused the formation of the Gd-rich phase shell on the ball surface. Inside the ball, the ball center-towards Marangoni migration of the Gd-rich droplets also causes the formation of a region with a lower (or even 0) number density of the droplets (region III in Fig. 1E). Thus, the Marangoni migration of droplets together with the diffusional transfer of solute Gd described above causes the formation of the Gd-poor region (region II + III) next to the Gd-rich phase shell. Enhancing the cooling rate causes a diminishment in the thickness of the Gd-rich phase shell and width of the Gd-poor region, and promotes the formation of a well-dispersed microstructure.
